# Dementia as a predictor of palliative care: Uncovering patient patterns based on German claims data

**DOI:** 10.1186/s12904-025-01672-y

**Published:** 2025-02-18

**Authors:** Elena Rakuša, Constantin Reinke, Gabriele Doblhammer, Lukas Radbruch, Matthias Schmid, Thomas Welchowski

**Affiliations:** 1 German Center for Neurodegenerative Diseases, 53127 Bonn, Germany; 2 Institute for Sociology and Demography, Faculty of Economics and Social Sciences, University Rostock, Ulmenstraße 69, 18057 Rostock, Germany; 3 Department of Palliative Medicine, University Hospital Bonn, 53127 Bonn, Germany; 4 Institute of Medical Biometry, Informatics and Epidemiology (IMBIE), Medical Faculty, University of Bonn, 53127 Bonn, Germany; 5 Institute of Psychology, Psychological Methods, Evaluation and Statistics, Department of Psychology, University of Zurich, Zurich, Switzerland

**Keywords:** Dementia, Palliative care, End-of-life, Predictions, Machine learning

## Abstract

**Background:**

Palliative care aims to ensure a dignified and self-determined life for people facing the end of life. While palliative care is established for tumor diseases, it’s notably absent from German medical guidelines for other progressive diseases with an unfavorable prognosis such as dementia. This study will identify predictors of palliative care use in older patients and explore how these predictors relate to the probability of palliative care.

**Methods:**

We used data from the largest German health insurance company of people over 50 years of age from the period 2014–2019. The analysis focused on the last year of life. Outcomes were outpatient and inpatient palliative care and predictors were demographics, comorbidities, therapeutic remedies and rehabilitation, care and medical interventions, medication and patient group. Combined logistic regression models and discrete conditional inference survival forests were used to predict the utilization of outpatient and inpatient palliative care. For evaluation we used concordance-index and calibration plots. We identified the most important predictors by using a permutation approach and the log-loss metric.

**Results:**

The study cohort for the analysis of inpatient palliative care comprised 43,896 patients, while the cohort for the analysis of outpatient palliative care included a total of 37,430 patients. The models had appropriate discriminatory power (inpatient palliative care: concordance-index = 0.737 (95%CI = 0.721–0.754); outpatient palliative care: concordance-index = 0.689; 95%CI = 0.675–0.704) and showed appropriate calibration. A diagnosis of dementia, like a diagnosis of cancer, is predictive of inpatient palliative care and outpatient palliative care. We observed a lower probability for inpatient and for outpatient palliative care for dementia patients compared to cancer patients.

**Conclusions:**

The findings highlight the need to focus palliative care on other patient groups besides cancer patients, such as dementia patients, and to facilitate access for all patients.

**Supplementary Information:**

The online version contains supplementary material available at 10.1186/s12904-025-01672-y.

## Background

Hospice and palliative care in Germany include multi-professional care for people with incurable diseases and a limited life expectancy. The focus is on maintaining quality of life and ensuring a dignified death, as well as the alleviation of pain or other symptoms [1–3]. Palliative care can be provided on an outpatient or inpatient basis. Depending on need and cost, a distinction is made between general outpatient palliative care and specialized outpatient palliative care [3–5]. General outpatient palliative care includes palliative care that can be provided by primary care providers (primarily office-based primary care physicians, specialists, and outpatient care services) with basic palliative care training [6]. Care includes primary care visits and nursing services to manage the signs and symptoms of the disease [2, 4, 5, 7]. Specialist palliative home care services are available for complex cases, for patients at home, in nursing homes or long-term care facilities [3]. It includes specialized medical and nursing care, social interaction, and often even logotherapy and physiotherapy [8]. Patients with advanced disease, severe and difficult-to-control symptoms requiring hospitalization are eligible for inpatient palliative care.

Palliative care focus on a wide range of serious and progressive diseases and affected organs. This includes cancer as well as advanced heart, lung, kidney, neurological diseases and multimorbid geriatric patients [2, 9].

In Germany, medical guidelines (S3-guidelines [8]) recommend that patients with incurable cancer should have access to palliative care, regardless of disease stage or therapy [8]. In addition, patients should be offered palliative care after a diagnosis of incurable cancer, regardless of the targeted therapy. While the integration of palliative care for tumor diseases is increasingly established in oncology and general health care in cases of progressive disease and unfavorable prognosis, palliative care is surprisingly not mentioned in guidelines for other progressive diseases, such as dementia [10].

Demographic aging is leading to an increase in the total number of older people with limitations due to age and disease, who require temporary or permanent support or care. As a consequence, long-term care and palliative care are becoming increasingly important in the context of health care [11–13], as elderly morbid patients are predominantly cared for at home by family or self-funded professional care services [11]. With outpatient palliative care, the patient can receive additional support at home [3]. If the family cannot provide the care, care can be shifted to the inpatient care setting. The patient may receive outpatient specialist palliative care in a nursing home or can admitted to an inpatient palliative care or hospice setting [3].

The rise in the elderly population will also lead to an increase in the number of people with dementia, as the strongest risk factor for developing dementia is advancing age [14, 15]. Dementia is often not perceived as a life-limiting condition that requires palliative care, although it is an incurable, progressive process in which the patient gradually loses the ability to manage daily life and independence, often requires assistance and care, and with to a higher mortality rate [9]. Two-thirds of dementia patients in Germany currently receive informal care at home. As the number of dementia patients increases, the number of people in nursing homes will also rise. Although the symptom burden is similar to that of cancer patients, many dementia patients do not receive adequate and timely care [16].

Due to the discrepancy between care recommendations, and the associated increase in the care needs of patients at the end of life, we aim to investigate the utilization of end-of-life healthcare at in this paper. We aimed to identify predictors of palliative care utilization among older people and then examine the probability of palliative care.

We hypothesized that a cancer diagnosis is an important predictor of palliative care and increases its probability independently from in- or outpatient palliative care. In contrast, a dementia diagnosis is a less important predictor and reduces the probability of palliative care in any setting. Other important predictors are age and long-term care.

## Methods

### Data

We used longitudinal data from Germany’s largest health insurance company, Allgemeine Ortskrankenkasse (AOK). The data sample was randomly drawn in 2014 from individuals aged 50 and older (*N* = 250,000) and we followed them quarterly from the beginning of 2014 until the end of 2019. The data include demographic information on gender, year and month of birth and death (if applicable), place of residence, and whether or not the person lived in an institution. In addition, the sample contains information on all inpatient and outpatient diagnoses, coded according to the German modification of the 10th revision of the International Statistical Classification of Diseases and Related Health Problems (ICD-10). The data included filled prescriptions for medications coded according to the Anatomical Therapeutic Chemical Classification of Active Substances and Drugs code (ATC) and surgeries based on the German procedure classification, a modification of the International Classification of Procedures in Medicine (ICPM) (OPS, https://www.dimdi.de/dynamic/en/classifications/ops/, accessed: June 1, 2024). The data were anonymized claims data that did not require ethical review or patient consent.

### Study design

To predict the probability of end-of-life palliative care, we selected individuals who died during the observation period and were observed for a one year prior to death (Supplementary Figure S1). In addition, skin cancer patients were excluded from the study group, as skin cancer can be detected and treated at an early stage through Germany’s statutory cancer screening program. As a result, morbidity and mortality are significantly lower than for other types of cancer [17]. Our study cohort consisted of 37,430 patients. Since the data was unbalanced in terms of inpatient palliative care cases, a minority over-sampling was done by random sampling with replacement to increase the number of cases to address this issue [18]. After up-sampling the observations, we had a study population of 43,896 for the inpatient palliative care analysis. For the outpatient palliative care analysis, we used the non-up-sampled study cohort of 37,430 patients.

We generated training, test, and validation data separately for outpatient and inpatient palliative care. To create training populations, we randomly selected 60% of each study cohort, stratified by palliative care (palliative care vs. no palliative care, see Sect. 2.3 for more details), time to diagnosis and patient groups (see Sect. 2.4 for more details). The remaining subjects each study cohort were equally divided into validation and test groups. We trained the models on training datasets and used the validation datasets to find the optimal parameter values. Finally, we fit the models with optimal parameter values to the test data.

### Outcomes

The outcome for outpatient palliative care (OPC) was a binary variable and included general outpatient palliative care and specialized palliative care, as well as palliative care (PC) in primary care. The outcome for inpatient palliative care (IPC) was also a binary outcome. The precise definitions of both are provided in Supplementary Table S1. Both outcomes were defined according to ICD-10, Einheitlicher Bewertungsmaßstab (EBM) and Operationen- und Prozedurenschlüssel (OPS). EBM is the uniform valuation scale for services provided by registered physicians and registered psychotherapists in Germany. It is a social security list in the German health care system according to which outpatient and physician services are covered by health insurance. OPS is an official classification for the systematic recording and coding of all operations, procedures and general medical measures.

### Exposure

We categorized patient status into six subgroups: dementia patients (ICD-10 codes: F00-F03, F05.1, G30, G31.0), cancer patients (ICD-10 codes: C00-C41, C45-C97), patients with a diagnosis of dementia and cancer, and patients without a dementia diagnosis and without a cancer diagnosis (in the following referred as non-dementia & non-cancer). The group with a diagnosis of cancer and diagnosis of dementia was further divided into three subgroups: patients who received a diagnosis of dementia and a diagnosis of cancer within 12 months (referred as cancer & dementia), patients who received a diagnosis of dementia first and a diagnosis of cancer at least 12 months apart (referred as dementia & subsequent cancer), patients who received a diagnosis of cancer first and a diagnosis of dementia at least 12 months apart (referred as cancer & subsequent dementia). All were included as time-dependent binary “ever” variables with a value of 1 starting from the first occurrence of a disease and zero otherwise. Patient status may change over time. For example, a patient may be in the non-dementia & non-cancer group at baseline and receive a diagnosis of dementia during the observation period. At this point, the patient moves to the patient status dementia patients and the old patient status variable (non-dementia & non-cancer) is reset to zero and the new patient status variable (dementia patients) is set to one. Thus, only one patient status group applies to a person at a time.

### Predictors

We included the following predictors and categorized them into five different groups. **Therapeutic remedies and rehabilitation**: physiotherapy, occupational therapy, speech therapy, other therapies (excluding physiotherapy, occupational therapy, and speech therapy), general practitioner (GP) visits, neurologist visits, doctors’ visits (without GP and neurologist) and rehabilitation. Further information can be found in Supplementary Table S2. **Medical interventions and major medications**: chemotherapy, nuclear therapy, radiotherapy, cancer and dementia drugs. **Comorbidities** defined by the Elixhauser index [19]: hypertension, diabetes, chronic pulmonary disease, congestive heart failure, depression, cardiac arrhythmias, fluid and electrolyte disorders, rheumatoid arthritis/collagen vascular disease, renal failure, valvular disease, peripheral vascular disease, coagulopathy, pulmonary circulated disorders, liver disease, obesity, paralysis, deficiency anemia, hypothyroidism, drug abuse, peptic ulcer disease, weight loss, AIDS/HIV, blood loss anemia, alcohol abuse and neurological disorders without dementia. All descriptions of the comorbidities can be found in Supplementary Table S3. Category **care interventions** included information on whether patients lived in a nursing home and on the presence or absence of severe care dependency, where severe care dependency was defined as care level 3 (until the end of 2016) or care level 5 (from 2017), IPC and OPC (according to model) and information on the number of hospital days. Further details on long-term care including definition on care levels are available in Supplementary Method S1. We also included **demographics** such as age at baseline and sex.

All predictors except age, sex, different physician visits, and number of hospital days, were included as time-dependent binary “ever” variables with a value of 1 starting from the first occurrence of a given code and zero otherwise.

### Statistical analysis

The longitudinal information of the patients was analyzed using discrete time-to-event analysis with time since start of observation in quarters as analysis time. Since all observations started one year before death the maximum number of observed quarters was four. To predict the probability of PC, we used a combination of a logistic regression model (glm) and discrete conditional inference survival forests (cforests) [20]. Glm and cforest predictions were predicted individually and then averaged without weighting. The input data for these models consisted of one row per quarter and patient with information on the utilization of IPC, OPC and time-dependent predictors (as described in Sect. 2.5). All calculations were done in R version 4.1.3 (2022-03-10). For cforest, we used the partykit package (version 1.2.16) [21] to perform separate models for OPC and IPC. In addition, we calculated separately logistic regression models and a conditional inference survival forests for both outcomes in a sensitivity analysis.

To indicate whether the predicted features in- or decrease the probability of PC, we calculated odds ratios (OR) for each outcome using the 20 most important features.

### Model evaluation

We calculated the concordance index (c-index) to assess discriminatory power using the discSurv package (version 2.0.0) [22], 95% confidence intervals (95% CI) were calculated using 1000 bootstrap replications from the test data. In addition, we examined calibration plots (to graphically assess model calibration) and calculated a logistic recalibration intercept and slope to assess whether predicted risks were systematically over- or underestimated. To maximize model performance, we used the validation data to adjusted the “mtry” parameter (the number of input variables randomly samples at each node available for splitting). Using a permutation approach and the log-loss metric, we identified the key features for prediction.

## Results

First, we examined whether our study sample reflected the use of PC at the end of life in Germany. For this purpose, we analyzed the development of the utilization rates for OPC and IPC (percentage of deceased insured persons with OPC or IPC out of all deceased insured persons in a given year). The use of OPC increased from 25.5 to 28.8% from 2015 to 2019. IPC use increased from 7.7 to 8.5%. The results of the utilization rates for OPC and IPC can be found in Supplement Figure S2.

### Study cohort

#### Study population for inpatient palliative care at baseline

Our study cohort consisted of 43,896 patients, of whom 20,015 (45.60%) were male and 23,881 (54.40%) female. At baseline the mean age was 78.62 years. 558 patients (1.27%) received IPC and about 20% of the patients were already living in a nursing home at baseline. (Table 1). A cancer diagnosis was documented for 21.72% of the study participants; 24.84% had a dementia diagnosis, 47.71% had neither dementia nor a cancer diagnosis. About 5% had a combination of dementia and cancer diagnosis. The most common comorbidities in the study group were diabetes (43.48%), congestive heart failure (42.33%), cardiac arrhythmias (39.54%) and renal diseases (35.71%). Dementia-related medications were prescribed to more than 40% of patients (Table 1). The average observation period was 2.94 quarters (one quarter minimum, four quarters maximum).

During the observation period, the distribution of patients between groups changed. At the beginning of the observation period, most patients were still non-dementia & non-cancer patients, but by the quarter of death, the largest group were the dementia patients with 32.79%. Non-dementia & non-cancer patients were the second largest group with 31.28%, followed by the cancer patients (24.77%). The proportion of the patients group cancer & dementia increased to 6.05%, the group dementia & subsequent cancer increased to 2.17%, and the group cancer & subsequent dementia increased to 2.95%. In subsequent quarters, the proportion of patients with an IPC prescription increased to a proportion of 11.89%. All results can be found in supplement Table S4.

#### Inpatient palliative care patients at prescription

At time of IPC prescription (*N* = 9,699), more than half (57.26%) were diagnosed with cancer (Table 1). Non-dementia & non-cancer patients were the second largest group, accounting for 18.79% of all IPC patients. Patients were often affected by disturbances in fluid and electrolyte balance (64.14%), peripheral vascular disorders (46.19%), congestive heart failure (44.95%), and peripheral vascular disorders s (44.36%). More than a third of patients (34.43%) were already receiving OPC.

**Table 1:** Characteristics of the study population at baseline and the time of prescription of IPC

	Study population for IPC at baseline (*N* = 43,869*)		IPC patients at prescription (*N* = 9,699)	
	N	%	N	%
**Outcome**				
Inpatient palliative care	558	1.27	9,699	100.00
**Patient group**				
Cancer	9,534	21.72	5,554	57.26
Dementia	10,902	24.84	1,297	13.37
Non-dementia & non-cancer	20,944	47.71	1,822	18.79
Cancer & dementia	1,752	3.99	578	5.96
Dementia & subsequent cancer	267	0.61	202	2.08
Cancer & subsequent dementia	497	1.13	246	2.54
**Demographics**				
Time since observation (mean)	4		3.93	
Age at baseline (mean)	78.62		74.83	
Men	20,015	45.60	4,697	48.43
**Comorbidities**				
Disturbances in fluid and electrolyte balance	14,872	33.88	6,221	64.14
Weight loss	4,894	11.15	3,144	32.42
Congestive heart failure	18,581	42.33	4,360	44.95
Peripheral vascular disorders	14,646	33.37	4,480	46.19
Cardiac arrhythmias	17,355	39.54	4,302	44.36
Renal disease	15,674	35.71	4,133	42.61
Depression	13,756	31.34	3,906	40.27
Diabetes	19,088	43.48	4,229	43.6
**Therapeutic remedies and rehabilitation**				
Doctor´s visits (without GP and neurologist) (mean)	3.72		4.98	
**Care interventions**				
Outpatient palliative care	2,161	4.92	3,339	34.43
Nursing home	8,537	19.45	1,753	18.07
**Medical interventions and major medications**				
Chemotherapy	1,927	4.39	1,579	16.28
Dementia drugs	18,504	42.15	4,281	44.14
Radiotherapy	525	1.2	813	8.38

**Fig. 1:**
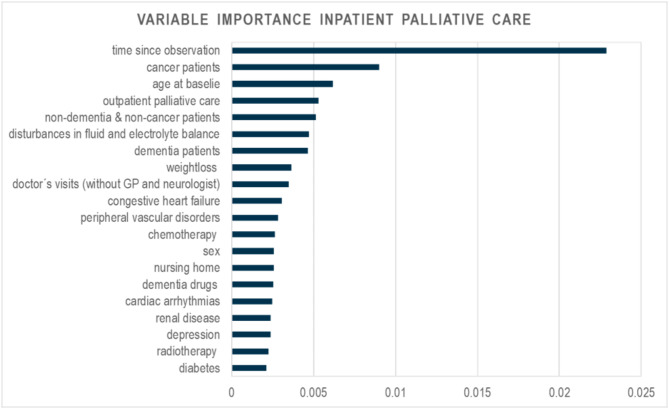
Variable importance for the model of inpatient palliative care. AOK data 2014–2019

We report only the 20 most influential predictors in terms of variable importance (Fig. 1). IPC: inpatient palliative care, * data up sampled. Data: AOK data from 2014–2019

#### Study population for outpatient palliative care at baseline

The dataset included a total of 37,430 patients. Of these, 48.77% had no dementia & no cancer diagnosis, 27.13% had a dementia diagnosis and 18.14% had a cancer diagnosis. Around 6% of patients had a cancer and dementia diagnosis (Table 2). The mean age at baseline was 79.28 years. The most common comorbidities with a frequency of over 30% were disturbances in fluid and electrolyte balance, depression and renal disease (Table 2). About 21% of all patients live in a nursing home and 8% receive long-term care. The average observation period was 2.94 quarters (one quarter minimum, four quarters maximum).

The distribution of patients among the groups changed during the observation period. The proportion of non-dementia & non-cancer patients decreased over the observation period from 48.77 to 34.87%, while the proportion of patients in all other patient groups increased. At the end of the observation period, 33.99% of the patients had a dementia diagnosis and 20.93% had a cancer diagnosis. 5.57% of the patients were cancer & dementia patients, and 1.85% of the patients were dementia & subsequent cancer patients. The proportion of cancer & subsequent dementia patients was 2.79%. At baseline, 4.31% of patients (*n* = 1,612) were already receiving OPC prescriptions. In subsequent quarters, the proportion of patients with a prescription varied. Only shortly before death the proportion of patients increased to 15.36% (quarter of death). All results can be found in supplement Table S5.

#### Outpatient palliative care patients at prescription

OPC patients (*N* = 9,992) were predominantly cancer patients (41.57%), followed by dementia patients (30.77%) (Table 2). Women were more likely to receive OPC (58.13%) and the most common comorbidities were disturbances in fluid and electrolyte balance (68.04%), renal disease (49.51%), and depression (42.96%). In addition, 11.19% of patients had previously received IPC.

**Table 2:** Characteristics of the study population at baseline and the time of prescription of OPC

	Study population for OPC at baseline (*N* = 37,430)		OPC patients at prescription (*N* = 9,992)	
	N	%	N	%
**Outcome**				
Outpatient palliative care	1,612	4.31	9,992	100.00
**Patient group**				
Cancer	6,791	18.14	4,154	41.57
Dementia	10,154	27.13	3,075	30.77
Non-dementia & non-cancer	18,256	48.77	1,288	12.89
Cancer & dementia	1,559	4.17	833	8.34
Dementia & subsequent cancer	237	0.63	333	3.33
Cancer & subsequent dementia	433	1.16	309	3.09
**Demographics**				
Time since observation (mean)	4		3.81	
Age at baseline (mean)	79.28		79.46	
Men	16,903	45.16	4,184	41.87
**Comorbidities**				
Weight loss	4,000	10.69	3,165	31.68
Disturbances in fluid and electrolyte balance	12,753	34.07	6,799	68.04
Depression	11,683	31.21	4,293	42.96
Paralysis	4,065	10.86	1,817	18.18
Renal disease	13,679	36.55	4,947	49.51
**Therapeutic remedies and rehabilitation**				
Doctor´s visits (without GP and neurologist) (mean)	3.68		5	
Physiotherapy	5,314	14.2	1,890	18.92
Rehabilitation	360	0.96	275	2.75
**Care interventions**				
Long-term care	3,001	8.02	2,349	23.51
Nursing home	8,000	21.37	3,852	38.55
Inpatient palliative care	186	0.5	1,118	11.19
**Medical interventions and major medications**				
Chemotherapy	1,169	3.12	1,047	10.48
Radiotherapy	330	0.88	362	3.62

**Fig. 2:**
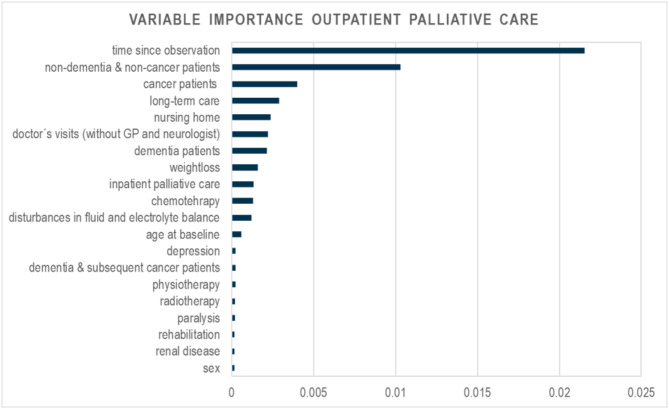
Variable importance for the model of outpatient palliative care. AOK data 2014–2019

We report only the 20 most influential predictors in terms of variable importance (Fig. 2). OPC: outpatient palliative care. Data: AOK data from 2014–2019

### Model evaluation

**Fig. 3:**
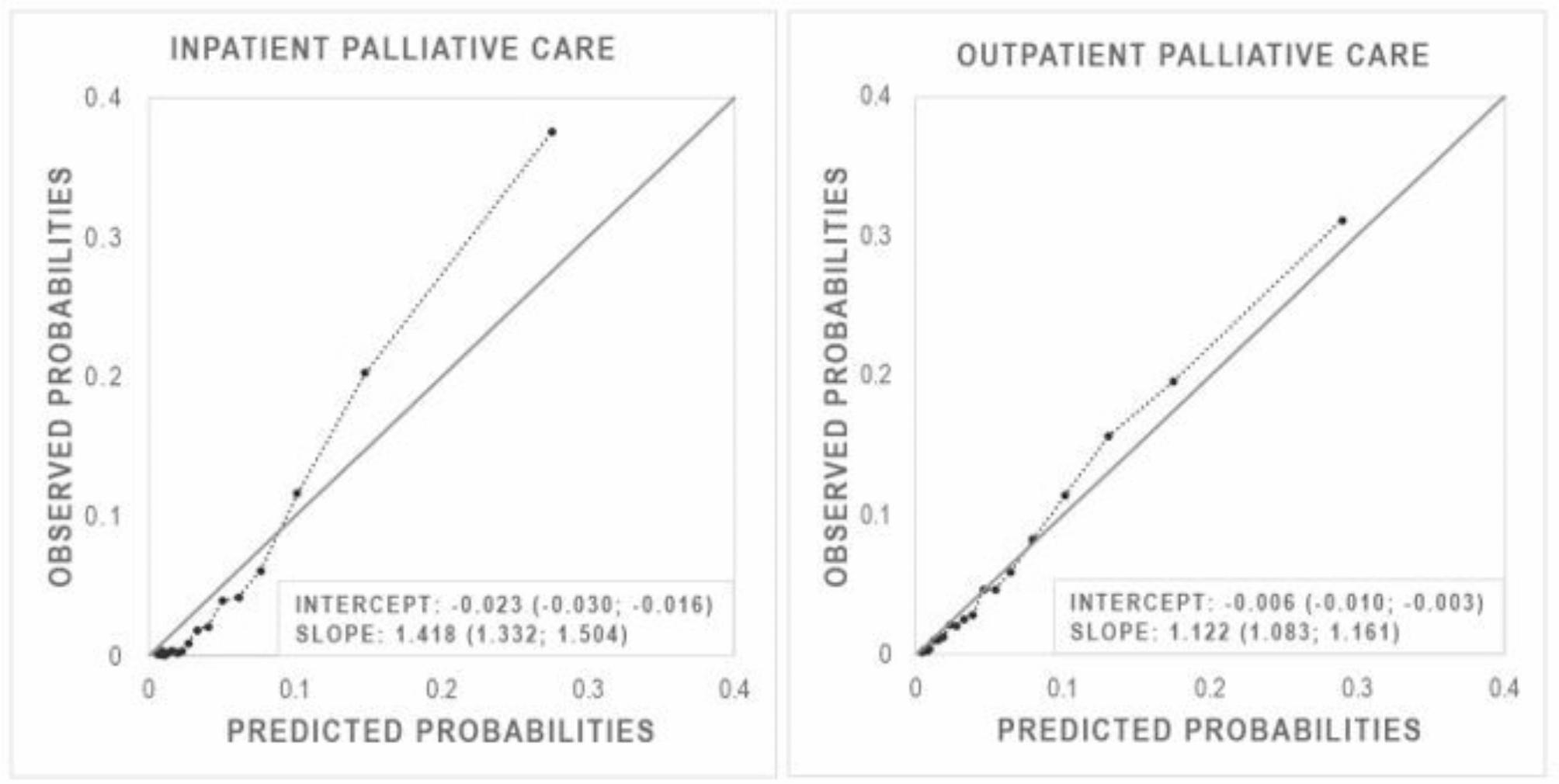
Calibration plots. Intercepts and slopes were calculated using logistic recalibration (95% confidence intervals in parentheses)

First, we used both a glm and a cforest to predict IPC as well as OPC. The cforest had a higher discriminatory power than the glm model for the prediction of IPC. However, the calibration of the cforest was worse than that of the glm model. All results are shown in Supplementary Table S6 and Figure S3. Therefore, we used a combined model of both approaches. The c-index was 0.737 (95% CI = 0.721–0.754), and the calibration was acceptable with an intercept of -0.023 (95% CI = -0.030 - -0.016) and a slope of 1.418 (95% CI = 1.332–1.504) (Fig. 3).

The discriminative power of glm and cforest for OPC was comparable. The glm model tended to slightly overestimate the predicted values, while the cforest had the opposite effect and tended to underestimate the predictions. All results are shown in Supplementary Table S6 and Figure S3. Again, we decided to combine both models to predict OPC. The c-index was 0.689 (95% CI = [0.675–0.704]), and the calibration plot showed that the combined model was better calibrated than the individual models, with intercept close to 0 and slope close to 1 (intercept: -0.006; 95% CI = -0.010 - -0.003; slope: 1.122; 95% CI = 1.083–1.161) (Fig. 3).

### Most important variables

#### Inpatient palliative care

The most important variable was the time since observation (Fig. 1). The second and third most important variables were cancer diagnosis and age at baseline. In addition to cancer diagnoses, non-dementia & non-cancer, and dementia diagnoses were among the top 20 predictors (3/6). Eight diseases from the comorbidities (disturbances in fluid and electrolyte balance, weight loss, congestive heart failure, peripheral vascular disorders, cardiac arrhythmias, renal disease, depression and diabetes) (8/28) and one therapeutic remedy and rehabilitation (number of doctor´s visits) (1/8) were among the most predictive factors. Three medical interventions and major medications (chemotherapy, dementia medications, radiotherapy) (3/5), and two care interventions (OPC and nursing home) (2/5) were also among the top 20 predictors.

The probability of prescribing IPC was primarily increased by characteristics of comorbidities, medical interventions and major medications (Supplement Figure S4). Patients who receive chemotherapy (OR = 1.51; 95% CI = 1.41–1.61), radiation therapy (OR = 1.10; 95% CI = 1.05–1.15), or dementia drugs (OR = 2.95; 95% CI = 2.69–3.24) had an increased probability of IPC compared to patients who did not receive any of these medical interventions or major medications (Table 3). With each quarter of observation, the probability of IPC increased. Women and patients who had already received OPC had a significantly increased probability of IPC (women: OR = 1.16, 95% CI = 1.11–1.22; OPC: OR = 1.65 95% CI = 1.56–1.73).

All patient groups had a significantly lower probability of receiving IPC compared to cancer patients (dementia: OR = 0.28, 95% CI = 0.26–0.30; non-dementia & non-cancer: OR = 0.30, 95% CI 0.20–0.32; cancer & dementia: OR = 0.61, 95% CI = 0.56–0.67; dementia & subsequent cancer: OR = 0.70, 95% CI = 0.60–0.82; cancer & subsequent dementia: OR = 0.58, 95% CI = 0.51–0.67).

**Table 3:** Odds ratios from logistic regression with inpatient palliative care as the outcome

Inpatient palliative care	Odds Ratio	*P* > z	[95% Conf. Interval]	
**Demographics**				
Time since observation	1.71	< 0.001	1.67	1.74
Age at baseline	0.99	< 0.001	0.98	0.99
Sex				
Male	Ref			
Female	1.16	< 0.001	1.11	1.22
**Patients group**				
Cancer patients	Ref.			
Dementia patients	0.28	< 0.001	0.26	0.30
Non-dementia & non-cancer patients	0.30	< 0.001	0.29	0.32
Cancer & dementia patients	0.61	< 0.001	0.56	0.67
Dementia & subsequent cancer patients	0.70	< 0.001	0.60	0.82
Cancer & subsequent dementia patients	0.58	< 0.001	0.51	0.67
**Comorbidities**				
Disturbances in fluid and electrolyte balance	1.35	< 0.001	1.29	1.42
Weight loss	1.35	< 0.001	1.28	1.42
Congestive heart failure	0.85	< 0.001	0.81	0.90
Peripheral vascular disorders	1.13	< 0.001	1.08	1.18
Cardiac arrhythmias	0.93	0.005	0.89	0.98
Renal disease	0.97	0.209	0.92	1.02
Depression	1.10	< 0.001	1.05	1.15
Diabetes	0.99	0.538	0.94	1.03
**Therapeutic remedies and rehabilitation**				
Doctor´s visits (without GP and neurologist)	1.01	< 0.001	1.01	1.01
**Care interventions**				
Outpatient palliative care	1.65	< 0.001	1.56	1.73
Nursing home	0.73	< 0.001	0.68	0.77
**Medical interventions and major medications**				
Chemotherapy	1.51	< 0.001	1.41	1.61
Dementia drugs	1.10	< 0.001	1.05	1.15
Radiotherapy	2.95	< 0.001	2.69	3.24

Controlled for the top 20 predictors. AOK data 2014–2019

#### Outpatient palliative care

The most important predictor was time since observation (Fig. 2). This was followed by non-dementia & non-cancer diagnosis and cancer diagnosis. In addition to these diagnosis dementia, cancer & dementia were predictive for OPC (4/6). The comorbidities weight loss, disturbances in fluid and electrolyte balance, depression, paralysis and renal disease (5/28) were predictive for OPC. In addition to time since observation, the demographic factors of age at baseline and sex were important predictors of OPC (3/3), as well as long-term care, IPC and nursing home from care interventions (3/5), doctors’ visits (without GP and neurologist), physiotherapy and rehabilitation from therapeutic remedies and rehabilitation (3/8), and chemotherapy and radiotherapy from medical interventions and major medications (2/5).

The probability of an OPC prescription was mainly influenced by the comorbidity structure of the patients, as well as therapeutic remedies, rehabilitation and care interventions (Supplement Figure S5). The OPC probability increased significantly with the use of physiotherapy (OR = 1.09; 95% CI = 1.03–1.15), rehabilitation (OR = 1.58; 95% CI = 1.38–1.82), chemotherapy (OR = 2.05; 95% CI = 1.89–2.23) and radiotherapy (OR = 1.57; 95% CI = 1.38–1.80) (Table 4). Patients with existing care intervention also had an increased probability of OPC compared to patients without these care (long-term care: OR = 1.93, 95% CI = 1.83–2.05; nursing home: OR = 1.49, 95% CI = 1.41–1.57; IPC: OR = 1.97, 95% CI = 1.82–2.13).

The probability of OPC was lower in all groups (except those with dementia & subsequent cancer diagnosis) compared to those cancer diagnosed (dementia: OR = 0.33; 95% CI = 0.31–0.36; non-dementia & non-cancer: OR = 0.18; 95% CI = 0.17–0.19; cancer & dementia: OR = 0.64; 95% CI = 0.59–0.70; and cancer & subsequent dementia: OR = 0.54; 95% CI = 0.48–0.62).

**Table 4:** Odds ratios from logistic regression without patient palliative care as the outcome

Outpatient palliative care	Odds Ratio	*P* > z	[95% Conf. Interval]	
**Demographics**				
Time since observation	1.46	< 0.001	1.44	1.49
Age at baseline	1.00	0.002	1.00	1.01
Sex				
Male	Ref.			
Female	1.12	< 0.001	1.07	1.17
**Patient groups**				
Cancer patients	Ref.			
Dementia patients	0.33	< 0.001	0.31	0.36
Non-dementia & non-cancer patients	0.18	< 0.001	0.17	0.19
Cancer & dementia patients	0.64	< 0.001	0.59	0.70
Dementia & subsequent cancer patients	1.00	0.954	0.88	1.15
Cancer & subsequent dementia patients	0.54	< 0.001	0.48	0.62
**Comorbidities**				
Weight loss	1.57	< 0.001	1.50	1.65
Disturbances in fluid and electrolyte balance	1.39	< 0.001	1.33	1.46
Depression	1.17	< 0.001	1.11	1.22
Paralysis	1.07	0.025	1.01	1.13
Renal disease	1.00	0.856	0.96	1.05
**Therapeutic remedies and rehabilitation**				
Doctor´s visits (without GP and neurologist)	1.03	< 0.001	1.03	1.03
Physiotherapy	1.09	0.002	1.03	1.15
Rehabilitation	1.58	< 0.001	1.38	1.82
**Care interventions**				
Long-term care	1.93	< 0.001	1.83	2.05
Nursing home	1.49	< 0.001	1.41	1.57
Inpatient palliative care	1.97	< 0.001	1.82	2.13
**Medical interventions and major medications**				
Chemotherapy	2.05	< 0.001	1.89	2.23
Radiotherapy	1.57	< 0.001	1.38	1.80

Controlled for the top 20 predictors. AOK data 2014–2019

## Discussion

A diagnosis of dementia was a significant predictor of the use or non-use of PC, along with cancer. Patients with dementia had a significantly lower probability of both IPC and OPC compared to patients with cancer. They even had the lowest probability of receiving IPC. This is in line with existing studies on the patient situation in PC, where cancer patients still represent the largest proportion of patients [23, 24]. Furthermore, our data reflect the use of PC at the end of life in Germany and confirm the findings of Ditscheid et al. [25].

### Inpatient palliative care

Time since observation were the most important factors predicting IPC. Even though early integration of PC has been proven to be beneficial for patients with progressive life-limiting diseases [26], in practice, the involvement of PC often occurs near death [27]. Dementia diagnoses are among the ten most important predictors of IPC. We had expected that patients with a dementia diagnosis would have a lower probability of IPC than patients with a cancer diagnosis. Our analyses validated this hypothesis, as all other patient groups in our study exhibited a significantly lower probability of IPC than cancer patients. In particular, the patient group without a combined cancer diagnosis (non-dementia & non-cancer as well as dementia) had a significantly lower probability. Surprisingly, the patient groups with a combined cancer diagnosis (cancer & dementia, dementia & subsequent cancer and cancer & subsequent dementia) also showed a significantly lower probability.

This may be explained by the fact that most old people with dementia live in private households as well as in nursing homes and residential care facilities [28]. Instead of using IPC facilities, they and their families and caregivers may prefer OPC [29], which allows them to stay in their familiar home surroundings until death. Dementia patients who received IPC are more likely to show signs of advanced dementia [30], characterized by symptoms such as disorientation, helplessness, and inability to perform activities of daily living [31], beyond the point at which patients could be cared for at home as outpatients. Another barrier to IPC may be accessibility. Due to a lack of guidelines and indications for PC for patients with dementia, many patients have difficulty accessing care [10].

Patients who had already received OPC had an increased probability of IPC. These patients represent an already morbid patient population with the prospect of premature death or need for assistance with daily living and self-care [2, 4]. In cases of severe acute symptoms that cannot be adequately controlled in an outpatient setting, the patient may be transferred to an inpatient setting where PC physicians and nurses use a variety of medical and nursing interventions to re-stabilize the patient and, if possible, even discharge the patient home [7].

### Outpatient palliative care

We hypothesized that a cancer diagnosis would be an important predictor of PC and a dementia diagnosis would be a less important predictor. Both diagnosis groups rank among the top predictors, but as expected, the probability of OPC was lower in dementia patients compared to cancer patients. Specialized OPC offers the possibility of receive high-quality OPC not only at home but also in institutions. In Germany, most people with early stages of dementia remain at home and are cared for by family members [30, 32], and almost half of patients with dementia are receive care at home until death [33]. It is difficult to determine the optimal point in time when PC for dementia patients should be initiated [9, 34, 35]. During the final stage of life, people with advanced dementia may encounter significant physical and psychological symptoms. Various outpatient medical and nursing interventions can alleviate symptoms and enhance wellbeing. Physicians and outpatient care services with basic training in PC provide appropriate end-of-life care to dementia patients [33]; however, such care is not yet implemented regularly for people diagnosed with dementia, so many patients struggle to receive appropriate end-of-life care [10].

The probability of OPC is driven by the comorbidity structure, therapeutic remedies and rehabilitation as well as care interventions of the patients. Patients who have previously received long-term care, lived in a nursing home, or received care in an IPC facility have a high probability of being dependent on assistance. It is not surprising, therefore, that these patients had an increased probability of needing OPC compared to those without prior care experience. When basic PC interventions as part of primary care are no longer sufficient to alleviate symptoms specialist OPC can provide additional support for both patients and their families and caregivers [3]. Patients receiving IPC are more likely to require OPC services although most patients die in hospital [36]. This may be related to the fact that patients who have been discharged from IPC and who may not have died during IPC are at a higher risk of requiring OPC. The fundamental objective of IPC in Germany is to alleviate the symptoms and therapeutic interventions associated with the disease, thereby stabilizing the patient’s condition and facilitating discharge home or transition to another care facility [36]. These patients continue to receive OPC after being discharged from IPC.

Lack of communication and knowledge deficits about PC as well as the stigma of PC as an intervention for the imminently dying remain as additional barriers that may impede access to palliative care [37].

Our finding that women are at higher more likely to receive PC is well known [38, 39] and may be due to a variety of influences, including gender roles, stress, lifestyle, and preventive health measures [39]. Women also have a higher risk of lacking a support system. Statistically, women live longer than men [40], so often the sick husband can be cared for by the wife, but if she becomes ill later, the husband has often already died and she is dependent on other support systems.

### Strength and limitations

The study benefits from large, comprehensive data collected from the inpatient and outpatient sectors including nursing homes in Germany, which increases the validity of the study conducted. The standardized survey reduces problems such as selection and recall bias. However, the data are primarily used for billing purposes, which limits the generalizability of the results, especially for individuals who did not visit a physician. Additionally, the AOK has a higher percentage of individuals with a low socioeconomic status compared to other statutory health insurers and even in comparison to private health insurers in Germany [41]. While these disparities could potentially affect both morbidity and the utilization of healthcare services, they can be partially attributed to the differing age distribution within the AOK population, which is older than the German population. When considering patients of the same age, the difference in the social structure of the AOK population is more pronounced in younger age groups than in older ones [42, 43]. Information on medical prescriptions is limited to the filling of the prescription, without information on the actual intake of the medication.

In addition, there were initial problems in tuning the models. The cross-entropy based log-loss metric was employed to measure prediction quality. Tuning regarding c-index resulted in loss of calibration. Therefore, a combined model of glm model and cforest was selected as a compromise between c-index and calibration model coefficients. It is worth noting that the fitted model is only marginally better than the untuned model in terms of the c-index and the calibration model coefficients. Possible data-related issues should be considered. Our dataset comprised a limited number of cases of IPC, thus we conducted an up-sampling beforehand to artificially increase the sample.

To incorporates time-variable switching conditions and integrates IPC and OPC could represent a promising future advancement. However, it is imperative to consider that patients in Germany have the option of transitioning between IPC and OPC. The development of models that take this switching into account is only possible on the condition that the order of prescriptions is clearly recognizable in the data. The nature of our data collection process, which is conducted on a quarterly basis, precludes the identification of the sequence in which prescriptions are made. Further research can consider the integration of IPC and OPC, including time-varying switching conditions.

The provision of PC in Germany is influenced by both socioeconomic and medical factors [44], but socioeconomic variables were not included in our data. The decision to provide support depends on the person in need of care, where they live, and their care needs and the caregiver [11]. In 2021, 84% of Germans in need of long-term care were receiving care at home from informal caregivers and professional care services [32]. Providing care at home for relatives results in financial losses for the family caregiver since they have to reduce their working hours that ensure income if they are below retirement age [45].

Socioeconomically disadvantaged individuals demonstrate an increased need for medical care compared to the general population [46]. Access to PC services depends on physician referrals, as referral patterns for PC are not standardized, depend on many patient and provider characteristics, and are not necessarily based on actual need [46]. Individuals with low social security often have limited access to health care services [46]. Further analyses of PC with other data are advised to adequately address socioeconomic factors in addition to medical factors.

## Conclusions

Our analyses suggest that a diagnosis of dementia, like a diagnosis of cancer, is predictive of both IPC and OPC, even though dementia patients appear to have a much lower chance to access palliative care. It seems that dementia patients are less likely to receive both IPC and OPC. Particular attention should be paid to dementia patients with pre-existing comorbidities and those who are already in contact with the care system (either through nursing and medical interventions or through therapeutic and rehabilitative interventions). Patients with pre-existing care needs, such as long-term care, nursing home residents and rehabilitants, appear to be more likely to receive PC. These patients may have already exhausted all care interventions outside of PC, so that symptom control and care are still possible only in the inpatient setting. Another group with a comparatively high probability of PC are older women, which may indicate a lack of informal social support in older age. Our findings underline the need to focus PC on other patient groups besides cancer patients, such as dementia patients, and to facilitate access for all patients.

## Electronic supplementary material

Below is the link to the electronic supplementary material.

Supplementary Material 1

## Data Availability

The data that support the findings of this study are available from Scientific Institute of the AOK (WIdO) but restrictions apply to the availability of these data, which were used under license for the current study, and so are not publicly available. Data are however available from the Scientific Institute of the AOK (WIdO) upon reasonable request and with permission.

## Abbreviations

AOK: Allgemeine Ortskrankenkasse
ICD-10: International Statistical Classification of Diseases and Related Health Problems, 10th revision
ATC: Anatomical Therapeutic Chemical Classification of Active Substances and Drugs code
ICPM: International Classification of Procedures in Medicine
SMOTE: Synthetic minority over-sampling technique
PC: Palliative care
OPC: Outpatient palliative care
IPC: Inpatient palliative care
EBM: Einheitlicher Bewertungsmaßstab
OPS: Operationen- und Prozedurenschlüssel
GP: General practitioner
GLM: Logistic regression model
Cforest: Discrete conditional inference survival forests
C-index: Concordance index
95% CI: 95% confidence intervals
OR: Odds ratio
