# Correction: Dementia as a predictor of palliative care: uncovering patient patterns based on German claims data

**DOI:** 10.1186/s12904-025-01898-w

**Published:** 2025-10-20

**Authors:** Elena Rakuša, Constantin Reinke, Gabriele Doblhammer, Lukas Radbruch, Matthias Schmid, Thomas Welchowski

**Affiliations:** 1 German Center for Neurodegenerative Diseases, Bonn, 53127 Germany; 2 Institute for Sociology and Demography, Faculty of Economics and Social Sciences, University Rostock, Ulmenstraße 69, Rostock, 18057 Germany; 3 Department of Palliative Medicine, University Hospital Bonn, Bonn, 53127 Germany; 4 Institute of Medical Biometry, Informatics and Epidemiology (IMBIE), Medical Faculty, University of Bonn, Bonn, 53127 Germany; 5 Institute of Psychology, Psychological Methods, Evaluation and Statistics, Department of Psychology, University of Zurich, Zurich, Switzerland

**Correction: BMC Palliat Care 24, 46 (2025)**

**https://doi.org/10.1186/s12904-025-01672-y**

Following publication of the original article [1], the author reported that the below statement should be updated from.

“Since the data was unbalanced in terms of inpatient palliative care cases, the synthetic minority over-sampling technique (SMOTE) was used to increase the number of cases to address this issue [18, 19].”

to

“Since the data was unbalanced in terms of inpatient palliative care cases, a minority over-sampling was done by random sampling with replacement to increase the number of cases to address this issue [1].”

A new reference will also be added:

1. William G. Cochran. Sampling Techniques: Wiley series in probability and mathematical statistics. 3rd ed. New York: John Wiley & Sons, Ltd; 1977.

The original article has been updated.

## References

[1] Rakuša E, Reinke C, Doblhammer G, et al. Dementia as a predictor of palliative care: uncovering patient patterns based on German claims data. BMC Palliat Care. 2025;24:46. 10.1186/s12904-025-01672-y. 39966759 10.1186/s12904-025-01672-y PMC11834269

