# Supplementary material for: Dementia as a predictor of palliative care: Uncovering patient patterns based on German claims data
Source: BMC Palliat Care. 2025 Feb 18;24:46. doi: 10.1186/s12904-025-01672-y (PMC11834269; doi:10.1186/s12904-025-01672-y)

Is dementia predictive of palliative care? Uncovering patient patterns in palliative care at the end of life with a focus on dementia using German claims data

Elena Rakuša^1,2^, Constantin Reinke^2^, Gabriele Doblhammer^1,2^, Lukas Radbruch^3^, Matthias Schmid^1,4^, Thomas Welchowski^4,5^

^1^ German Center for Neurodegenerative Diseases, Bonn, Germany;
^2^ University Rostock, Rostock, Germany;
^3^ Department of Palliative Medicine, University Hospital Bonn, Bonn, Germany;
^4^ Institute of Medical Biometry, Informatics and Epidemiology (IMBIE), Medical Faculty, University of Bonn, Bonn, Germany
^5^ Institute of Psychology, Psychological Methods, Evaluation and Statistics, Department of Psychology, University of Zurich, Switzerland

Supplement

Figure S1: Flowchart of the analysis sample of inpatient and outpatient palliative care.

Table S1: Definition of the outcomes.

Table S2: List of ICD-10 codes of the disease.

Table S3: List of codes of the health services.

Method S1: Long-term care dependency.

Figure S2: Developing palliative care utilization rate over time (2015 to 2019).

Table S4: Number of patients within patient groups during the observation time for inpatient palliative care. Data: AOK data from 2014 - 2019. Own calculations.

Table S5: Number of patients within patient groups during the observation time for outpatient palliative care. Data: AOK data from 2014 - 2019. Own calculations.

Table S6: C-index for logistic regression models and cforest models.

Figure S3: Calibration plots for log. regression models and ctree models. Intercepts and slopes were calculated using logistic recalibration (95% confidence intervals in parentheses). Left column: Models with inpatient palliative care as outcome. Right column: Models with outpatient palliative care as outcome.

Figure S4: Number of top 20 features by category of variables associated with inpatient palliative care. AOK data 2014-2019.

Figure S5: Number of top 20 features by category of variables associated with outpatient palliative care. AOK data 2014-2019.

Figure S1: Flowchart of the analysis sample of inpatient and outpatient palliative care.


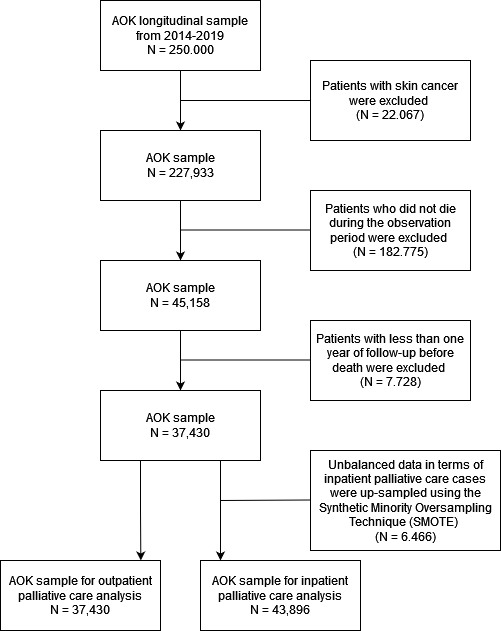


Table S1: Definition of the outcomes.

|  | **Outcome** | **Type of outcome** | **Type of palliative care included** | **Classification** |
| --- | --- | --- | --- | --- |
| Model 1 | outpatient palliative care | binary | general outpatient palliative care, specialized palliative care, palliative care in primary care | EBM: 03370-03373, 37300, 37302, 37305, 37306, 37314, 37317, 37318, 37320, 37400, 01425, 01426  ICD-10: Z51.5 |
| Model 2 | inpatient palliative care | binary | inpatient palliative care | OPS: 8982, 898h, 898e |

Table S2: List of codes of the health services.

|  | HeilM-RL | ICD-10 | EBM | OPS | ATC |
| --- | --- | --- | --- | --- | --- |
| **Therapeutic remedies and rehabilitation** |  |  |  |  |  |
| Physiotherapy | P |  |  |  |  |
| Occupational therapy | E |  |  |  |  |
| Speech therapy | L |  |  |  |  |
| Other therapies (excluding physiotherapy, occupational therapy, and speech therapy) | F |  |  |  |  |
| Rehabilitation |  | Z50.x | 01611,01622, 01623 |  |  |
| **Medical interventions and major medications** |  |  |  |  |  |
| Chemotherapy |  | Z51.1, Z51.2 |  | 854 |  |
| Nuclear therapy |  |  |  | 853 |  |
| Radiotherapy |  | Z510 |  | 852 |  |
| Cancer drugs |  |  |  |  | L01A, L01B, L01C, L01D, L01E, L01F, L01X |
| Dementia drugs |  |  |  |  | C03C,  C09D,  D03D, M05B,  N03A, N05A, N06A, N06D |
| HeilM-RL: Heilmittel-Richtlinie; ICD-10: German modification of the 10th revision of the International Statistical Classification of Diseases and Related Health Problems; EBM: Einheitlicher Bewertungsmaßstab; OPS: Operationen- und Prozedurenschlüssel; ATC: Anatomical Therapeutic Chemical Classification of Active Substances and Drugs code | | | | | |

Table S3: List of ICD-10 codes of the comorbidities.

| **Disease** | **ICD-10 Code** |
| --- | --- |
| Congestive heart failure | I09.9, I11.0, I13.0, I13.2, I25.5, I42.0, I42.5 - I42.9, I43.x, I50.x, P29.0 |
| Cardiac arrhythmias | I44.1 - I44.3, I45.6, I45.9, I47.x - I49.x, R00.0, R00.1, R00.8, T82.1, Z45.0, Z95.0 |
| Valvular disease | A52.0, I05.x - I08.x, I09.1, I09.8, I34.x - I39.x, Q23.0 - Q23.3, Z95.2 - Z95.4 |
| Pulmonary circulation disorders | I26.x, I27.x, I28.0, I28.8, I28.9 |
| Peripheral vascular disorders | I70.x, I71.x, I73.1, I73.8, I73.9, I77.1, I79.0, I79.2, K55.1, K55.8, K55.9, Z95.8, Z95.9 |
| Hypertension | I10.x, I11.x - I13.x, I15.x |
| Paralysis | G04.1, G11.4, G80.1, G80.2, G81.x, G82.x, G83.0 - G83.4, G83.9 |
| Other neurological disorders | G10.x - G13.x, G20.x - G22.x, G25.4, G25.5, G31.2, G31.8, G31.9, G32.x, G35.x - G37.x, G40.x, G41.x, G93.1, G93.4, R47.0, R56.x |
| Chronic pulmonary disease | I27.8, I27.9, J40.x - J47.x, J60.x - J67.x, J68.4, J70.1, J70.3 |
| Diabetes | E10.x, E11.x, E12.x, E13.x, E14.x |
| Hypothyroidism | E00.x - E03.x, E89.0 |
| Liver disease | B18.x, I85.x, I86.4, I98.2, K70.x, K71.1, K71.3 - K71.5, K71.7, K72.x - K74.x, K76.0, K76.2 - K76.9, Z94.4 |
| Peptic ulcer disease, excluding bleeding | K25.7, K25.9, K26.7, K26.9, K27.7, K27.9, K28.7, K28.9 |
| AIDS/HIV | B20.x - B22.x, B24.x |
| Rheumatoid arthritis/collagen vascular diseases | L94.0, L94.1, L94.3, M05.x, M06.x, M08.x, M12.0, M12.3, M30.x, M31.0 - M31.3, M32.x - M35.x, M45.x, M46.1, M46.8, M46.9 |
| Coagulopathy | D65 - D68.x, D69.1, D69.3 - D69.6 |
| Obesity | E66.x |
| Weight loss | E40.x - E46.x, R63.4, R64 |
| Fluid and electrolyte disorders | E22.2, E86.x, E87.x |
| Blood loss anemia | D50.0 |
| Deficiency anemia | D50.8, D50.9, D51.x - D53.x |
| Alcohol abuse | F10, E52, G62.1, I42.6, K29.2, K70.0, K70.3, K70.9, T51.x, Z50.2, Z71.4, Z72.1 |
| Drug abuse | F11.x - F16.x, F18.x, F19.x, Z71.5, Z72.2 |
| Psychoses | F20.x, F22.x - F25.x, F28.x, F29.x, F30.2, F31.2, F31.5 |
| Depression | F20.4, F31.3 - F31.5, F32.x, F33.x, F34.1, F41.2, F43.2 |

Method S1: Long-term care dependency.

Long-term care was defined as claiming benefits from the German statutory long-term care insurance. From the introduction of long-term care insurance in 1995 until the end of 2016, care status was divided into three care levels. The classification was based on the time required for daily assistance, so that patients in level one required the least effort and patients in level three required the most effort.

Since 2017, the three care levels were replaced by five care grades. The classification is no longer based on the time required for daily assistance, but is measured by how independently the person in need of care can manage their daily life. The degree of care increases with the severity of the impairment in everyday life.

For our analyses, we created a dummy variable to record the long-term care, which indicates the presence or absence of severe care dependency, so patients up to and including 2016 with care level 3 or from 2017 with care grade 5. All persons with care level 1 or 2 or care degree 1 to 4 are referred as "without severe long-term care".

Figure S2: Developing palliative care utilization rate over time (2015 to 2019).


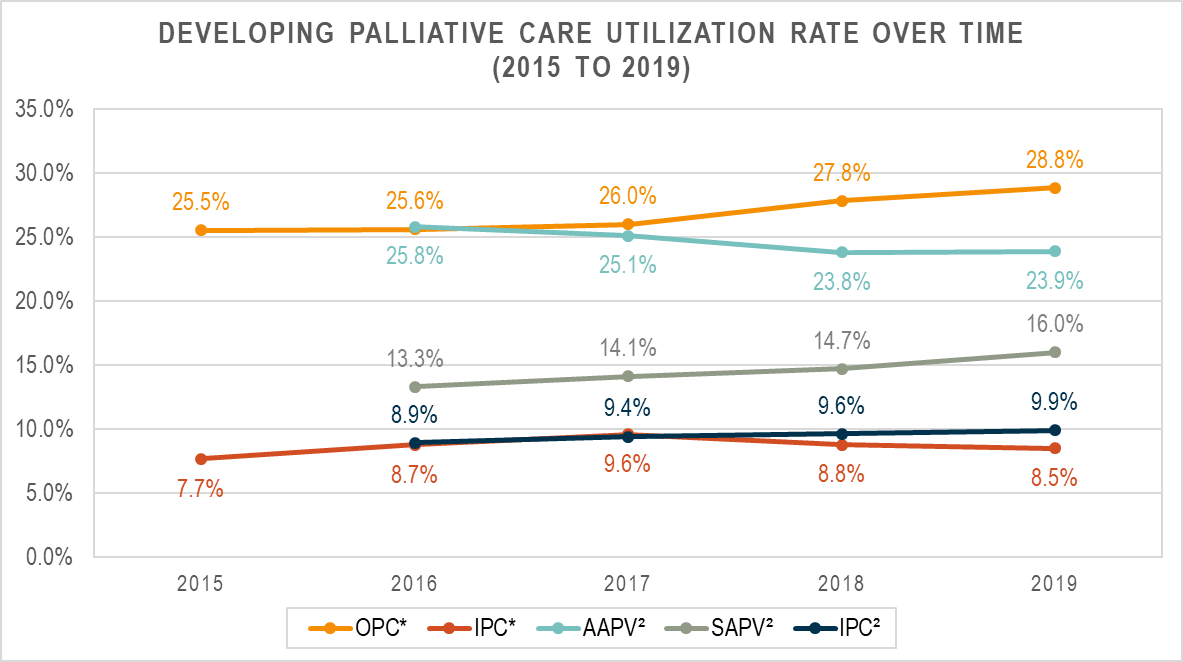


OPC: Outpatient palliative care; IPC: Inpatient palliative care; * Utilization rates from AOK Data; ² Utilization rates from Ditscheid et al.

Table S4: Number of patients within patient groups during the observation time for inpatient palliative care. Data: AOK data from 2014 - 2019.

|  | | inpatient palliative care | | cancer patients | | dementia patients | | non-dementia & non-cancer patients | | cancer & dementia patients | | dementia & subsequent cancer patients | | cancer & subsequent dementia patients | |
| --- | --- | --- | --- | --- | --- | --- | --- | --- | --- | --- | --- | --- | --- | --- | --- |
| quarter | N | N | % | N | % | N | % | N | % | N | % | N | % | N | % |
| 4 | 43,896 | 558 | 1.27 | 9,534 | 21.72 | 10,902 | 24.84 | 20,944 | 47.71 | 1,752 | 3.99 | 267 | 0.61 | 497 | 1.13 |
| 3 | 43,338 | 1,017 | 2.35 | 9,794 | 22.60 | 11,318 | 26.12 | 19,467 | 44.92 | 1,841 | 4.25 | 333 | 0.77 | 585 | 1.35 |
| 2 | 42,321 | 1,557 | 3.68 | 9,934 | 23.47 | 11,745 | 27.75 | 17,545 | 41.46 | 1,955 | 4.62 | 420 | 0.99 | 722 | 1.71 |
| 1 | 40,764 | 1,953 | 4.79 | 9,906 | 24.30 | 12,246 | 30.04 | 14,911 | 36.58 | 2,185 | 5.36 | 610 | 1.50 | 906 | 2.22 |
| quarter of death | 38,811 | 4,614 | 11.89 | 9,613 | 24.77 | 12,725 | 32.79 | 12,139 | 31.28 | 2,349 | 6.05 | 841 | 2.17 | 1,144 | 2.95 |

Table S5: Number of patients within patient groups during the observation time for outpatient palliative care. Data: AOK data from 2014 - 2019.

|  | | outpatient palliative care | | cancer patients | | dementia patients | | non-dementia & non-cancer patients | | cancer & dementia patients | | dementia & subsequent cancer patients | | cancer & subsequent dementia patients | |
| --- | --- | --- | --- | --- | --- | --- | --- | --- | --- | --- | --- | --- | --- | --- | --- |
| quarter | N | N | % | N | % | N | % | N | % | N | % | N | % | N | % |
| 4 | 37,430 | 1,612 | 4.31 | 6,791 | 18.14 | 10,154 | 27.13 | 18,256 | 48.77 | 1,559 | 4.17 | 237 | 0.63 | 433 | 1.16 |
| 3 | 35,818 | 607 | 1.69 | 6,485 | 18.11 | 10,103 | 28.21 | 17,011 | 47.49 | 1,486 | 4.15 | 254 | 0.71 | 479 | 1.34 |
| 2 | 35,211 | 843 | 2.39 | 6,633 | 18.84 | 10,442 | 29.66 | 15,686 | 44.55 | 1,562 | 4.44 | 305 | 0.87 | 583 | 1.66 |
| 1 | 34,368 | 1,951 | 5.68 | 6,921 | 20.14 | 10,833 | 31.52 | 13,728 | 39.94 | 1,695 | 4.93 | 458 | 1.33 | 733 | 2.13 |
| quarter of death | 32,417 | 4,979 | 15.36 | 6,785 | 20.93 | 11,020 | 33.99 | 11,303 | 34.87 | 1,805 | 5.57 | 601 | 1.85 | 903 | 2.79 |

Table S6: C-Index for glm models and cforest models.

|  | glm model | cforest model |
| --- | --- | --- |
| IPC | 0.723 (95% CI [0.705 - 0.740]) | 0. 741 (95% CI [0.725 - 0.756]) |
| OPC | 0. 687 (95% CI [0.673 - 0.702]) | 0. 682 (95% CI [0.666 – 0.697]) |
| IPC: inpatient palliative care; OPC: outpatient palliative care; Glm: logistic regression model; cforest: conditional inference survival forests | | |

Figure S3: Calibration plots for log. regression models (glm) and cforest models. Intercepts and slopes were calculated using logistic recalibration (95% confidence intervals in parentheses). Left column: Models with inpatient palliative care as outcome. Right column: Models with outpatient palliative care as outcome.


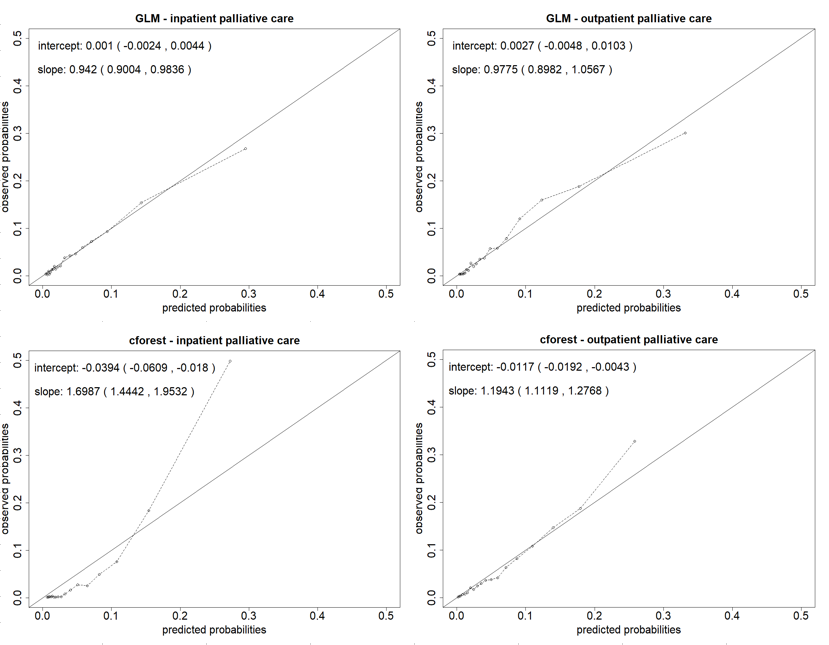


Figure S4: Number of top 20 features by category of variables associated with inpatient palliative care. AOK data 2014-2019.


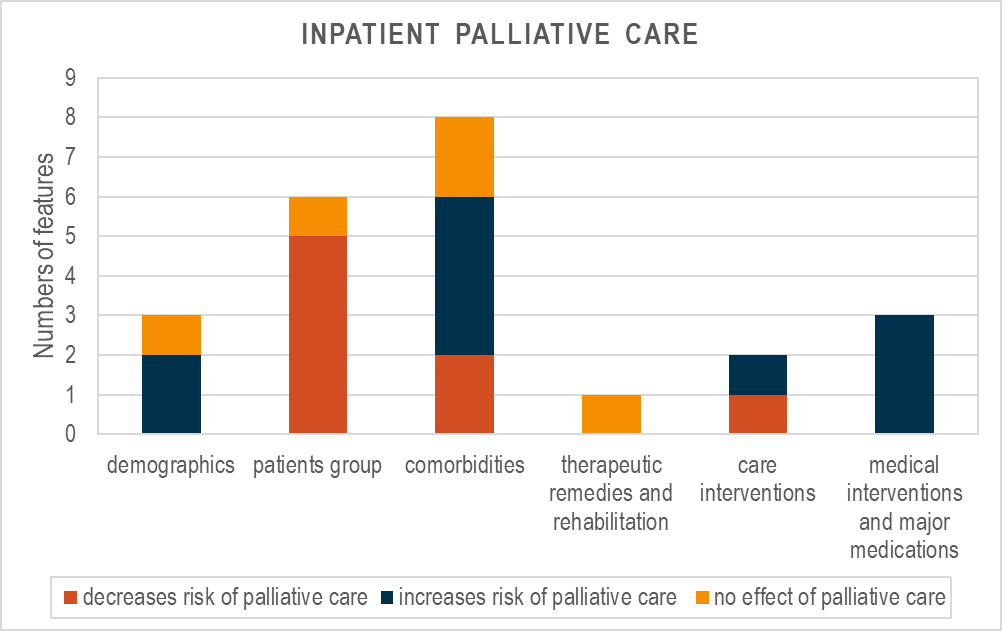


Figure S5: Number of top 20 features by category of variables associated with outpatient palliative care. AOK data 2014-2019.


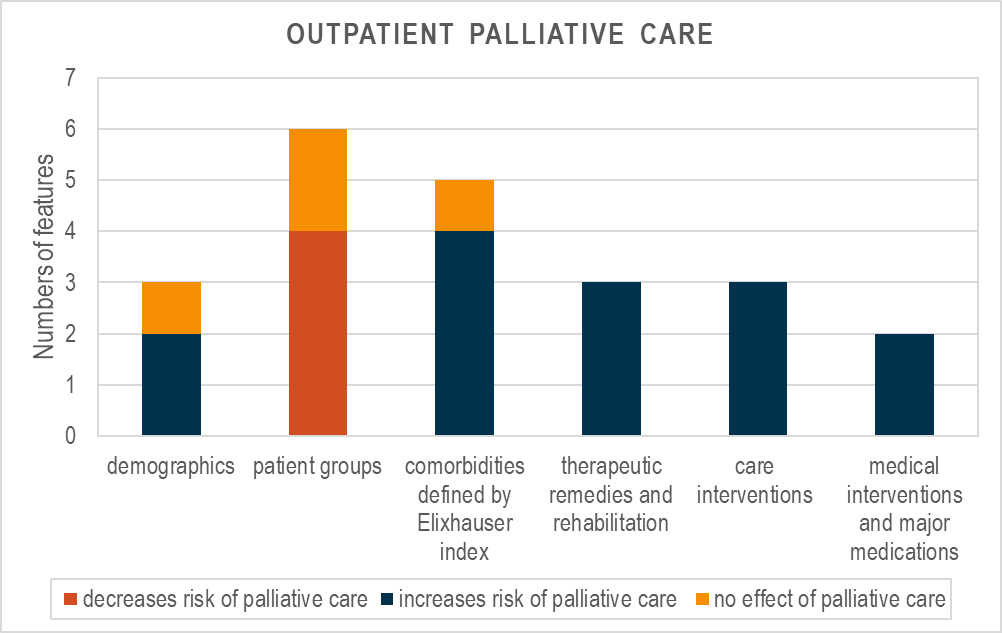

Supplement: Supplementary file 1 — Supplementary Material 1 [file 12904_2025_1672_MOESM1_ESM.docx]
